# Person-Centered Interactive Self-Management Support in Primary Health Care for People with Type 2 Diabetes: Protocol for a Randomized Controlled Trial

**DOI:** 10.2196/10250

**Published:** 2019-04-08

**Authors:** Ulrika Öberg, Ulf Isaksson, Lena Jutterström, Carljohan Orre, Åsa Hörnsten

**Affiliations:** 1 Department of Nursing Umeå University Umeå Sweden; 2 Department of Computer Science and Media Technology Malmö University Malmö Sweden

**Keywords:** type 2 diabetes, eHealth, internet, mobile apps, nurse specialists, primary health care, self-management, randomized controlled trial

## Abstract

**Background:**

Type 2 diabetes (T2D) is increasing as the population ages. The development of new medical treatments is promising and important, but the basic treatment remains self-management, even if adherence to lifestyle advice is low. Electronic health (eHealth) or mHealth interventions can increase empowerment among people living with T2D and may compensate for the lack of professional resources and geographical distances. The interactive self-management support (iSMS) project aims at including digital tools to support people living with T2D in their self-management and facilitating their interaction with diabetes specialist nurses (DSNs). This protocol outlines a study with the purpose of developing and evaluating an intervention where people living with T2D can increase self-efficacy and empowerment through digital self-monitoring and interaction with DSNs.

**Objective:**

To develop and evaluate a person-centered iSMS intervention in primary health care for people with T2D in addition to their usual diabetes care.

**Methods:**

This study is a 12-month, 3-armed, nonblinded randomized controlled trial (RCT), which will be conducted in 6 primary health care centers (HCCs) in northern Sweden. Eligible participants will be randomized to either an intervention group (n=46), a control group (n=46), or an external group (n=46) for comparison. The intervention group will receive the mobile app, and the control group will receive a minimal intervention (diabetes brochure) and the usual standard of care. Changes in glycated hemoglobin (HbA_1c_) will be the primary outcome measure.

**Results:**

This trial is currently open for recruitment. The first results are expected to be submitted for publication in Autumn 2019.

**Conclusions:**

This study, with its focus on iSMS, will provide insights regarding suitable ways to promote and develop a person-centered intervention. If successful, the intervention has the potential to become a model for the provision of self-management support to people with T2D.

**International Registered Report Identifier (IRRID):**

DERR1-10.2196/10250

## Introduction

### Background

Type 2 diabetes (T2D) occurs in up to 20% of people aged ≥70 years. T2D is a progressive disease with an increased risk of cardiovascular disease, cancer, and dementia. Physical inactivity and being overweight owing to an unhealthy lifestyle, such as that involving the use of tobacco and alcohol consumption, are key factors in the progression of T2D [1,2]. T2D requires both active self-management of people living with the disease and advanced medical treatment over time [1,2,3]. About 350,000 people living in Sweden are diagnosed with T2D [3], and the risk increases with age, regardless of gender [2,3]. Education levels relate to diabetes development, the higher the education, the lower the incidence. The incidence also varies by country (of birth) with higher prevalence among people born in the Nordic countries than in Europe outside Scandinavia [3].

Caring for people with T2D is a challenge for society, especially in rural areas that suffer from a shortage of general practitioners in primary health care [4]. In Sweden, primary health care nurses with responsibility for diabetes clinics (diabetes specialist nurses, DSNs) have a heavy workload and are often responsible for a large number of T2D patients regarding self-management support (SMS) and follow-ups. SMS includes motivating T2D patients to quit smoking, increase physical activity, change diet, initiate weight loss, and adhere to medication and blood sugar testing [5], all examples of frequent work tasks that fall on DSNs. The medical treatment is multifactorial and includes monitoring of blood sugar targets, blood pressure (BP), blood lipids, and other medical measures [6]. Furthermore, preventive measures such as retinal scans [7] and annual foot examinations are of great importance for delaying complications [8].

T2D is a complex disease with complex treatment, in which self-management is the basic treatment [9-11]. In recent years, SMS in groups and culturally appropriate education have been recommended, led by staff with both subject expertise and pedagogical training [3]. Findings in studies [10,12] show that SMS or patient education in groups and directed to individuals are equally effective among people with T2D and result in similar improvements in learning, behavioral, and clinical outcomes. In diabetes care, there have to be various individual options and scope for those who do not want to participate in or fit into groups. Additionally, person-centered care (PCC) that enables custom solutions and person-centered approaches that strengthen self-efficacy and patient empowerment are beneficial [10,13-14]. Electronic health (eHealth) interventions for chronically ill patients, instead of or in addition to usual care, can lead to positive effects on primary health outcomes [15]. eHealth interventions are also requested today and are motivated not only by the problems of geographical distance [16] and lack of health care staff [17] but also by the opportunities offered by the approach, namely, strengthened power and ownership as well as increased person-centeredness. Therefore, we believe that it is important to develop SMS using an eHealth intervention.

Recent research suggests that patients view the use of smartphone apps for self-monitoring and channels aimed at social support and interaction with the caregiver via computers and smartphones [18] as a good and important complement to traditional care. Some patients, however, express doubt about the technological issues that may arise [19]. A challenge may also be health care staff’s hesitation to use eHealth and mobile health in patient care. A recent interview study [20] among nurses in primary health care indicates that they viewed the trend toward eHealth approaches in patient care as unavoidable. However, the transition from traditional face-to-face visits to eHealth support could lead to a lack of control in their daily work, and they expressed a need to protect both themselves and the patients in the digital chaos created [20,21]. They preferred to meet patients face-to-face and saw a risk in the ongoing role change that may lead to losing their expert role in providing practical advice to patients. The solution could be to involve patients with T2D and DSNs in working together to develop an intervention with both obstacles and opportunities within their respective perspectives in mind. Several researchers recommend this kind of co-design and participatory design [22-24] since the implementation of new ideas is facilitated; these become accepted by users and are thereby longer-term solutions.

The purpose of the interactive self-management support (iSMS) project is to include digital tools by offering the use of a smartphone or tablet app to support people living with T2D in self-management and to facilitate interaction with DSNs. This study protocol outlines a randomized controlled trial (RCT) to evaluate the effectiveness of person-centered iSMS in primary health care. This study puts a particular focus on how digital technology is used as a tool for self-management in daily life, wherein patients with T2D manage their illness in closer collaboration with DSNs through self-monitoring and self-care activities. The objectives of this project are to develop and evaluate a person-centered iSMS intervention in primary health care for people with T2D in addition to their usual diabetes care. The hypotheses are that an iSMS program will decrease glycated hemoglobin (HbA_1c_) and improve metabolic measurements, such as BP (mm HG), body mass index, waist circumference (cm), and total and high-density lipoprotein cholesterol (mmol/L). Furthermore, we hypothesize that an iSMS program will improve lifestyle habits such as physical activity, diet, and smoking; decrease the need for SMS and changes in medical treatment; increase diabetes empowerment; increase diabetes-dependent quality of life (QoL); improve illness perception; and improve eHealth literacy in the intervention group compared with an internal (intervention and control) and external control group at 4 and 12 months’ follow-up.

### Theoretical Framework

This randomized intervention study is grounded on the theoretical perspectives of PCC, which is a care model that supports the person’s views about their life situation and condition as being indisputable and is always at the center of care. According to PCC, patients are persons who are more than their illness. PCC is based on the patient’s experience of the situation and the individual’s circumstances, resources, and obstacles. PCC has been described as the gold standard of care that will help individuals to develop the knowledge, skills, and confidence they need to more effectively manage and make informed decisions about their health or illness and health care and thus become partners in care. It also means that the patient should always be treated with respect. PCC is a partnership between patients or relatives and professional caregivers. It requires that health care professionals and patients together lay the ground for a relationship or partnership. The starting point for PCC is the patient’s story, which should be written in a structured way into a health plan that includes the goals and strategies for implementation and short- and long-term follow-up [25,26]. By shifting from an illness focus to a strengths-based, person-centered one, this intervention may change the usual care for people with T2D in primary health care. The process is intended to develop and implement an actionable plan to assist people with T2D in achieving their personal goals in the “illness process”. This RCT is intended to address the specific barriers that interfere with the person’s personal goal achievement; the aims are to transform and offer the participants a process for sustainable behavior change to fulfill their personal goals.

## Methods

### Trial Design

This protocol describes a 3-armed, nonblinded RCT to evaluate the effectiveness of person-centered iSMS in primary health care on metabolic balance, as measured by HbA_1c_. Within this project, interactive SMS (iSMS) is defined as person-centered and interactive self-management support. The person-centered part lies in an assessment of individual needs for SMS through a quantitative measurement and by listening to patients’ stories. The interactive part lies in the patients’ use of a smartphone or tablet app for self-monitoring and also interaction with the nurse and other participants through a patient forum when needed. Furthermore, other digital sources, such as a website with information about T2D, self-care, and so forth, will be included in the intervention. This protocol was prepared according to the Consolidated Standards of Reporting Trials [27], the Consolidated Standards of Reporting Trials extension for Electronic and mobile Health Applications and onLine TeleHealth interventions [28], and the Standard Protocol Items: Recommendations for Intervention Trials guidelines [29,30].

### Framework of Activities

#### Assessment of Patients’ Perceived Needs for Self-Management Support

A 10-item questionnaire, the Self-management Assessment Scale (SMASc), assessing patients’ needs for SMS, has been developed within the project, and its validity and reliability have been tested and found acceptable (manuscript, Öberg et al, 2018, unpublished data). The questionnaire is a person-centered measure of the type of iSMS that is needed for each person. The questionnaire includes subscales knowledge, routines, goals, emotional support, and social support and is generated from the literature on patient perspectives on chronic illness or T2D and self-management challenges.

#### Co-Designed Workshop

Several activities to prepare for the co-design and participatory workshop have been completed. Individual interviews among persons with T2D treated in Swedish primary health care centers (HCCs) were conducted to gain an understanding of their perceptions about and experiences of using eHealth services for self-management [19]. Furthermore, focus group interviews have been conducted with primary health care nurses about their perceptions of working with digital resources and iSMS in the care of people with chronic conditions, including T2D [20]. These earlier studies have been the basis for the development of the intervention.

A multistakeholder workshop was held on 16 September, 2016, which 27 invited participants attended, (5 were academic representatives, 6 were living with T2D, 2 were relatives of persons living with T2D, and 1 was a medical doctor and also the head of primary health care in the County Council). Furthermore, 10 were DSNs, 1 worked with information technology development in the County Council health care service and lastly, 2 were representatives of a Swedish company that develops apps for people living with diabetes.

The purpose of the workshop was to involve various participants in ideas about the design of the app, thereby influencing and developing the intervention and choice of app. During the workshop, the potentials and limitations of SMS with digital technology were explored and how SMS could be designed to motivate self-management in everyday life, at work and in the patients’ daily life with diabetes, was considered. The workshop used both focus group discussions with mixed groups of representatives and mentometers to answer questions. During the workshop, it was revealed that the most important needs were related to person-centeredness, accessibility, and effectiveness. The summary of the results from the workshop suggested guidelines for setting up the intervention (manuscript, Schimmer et al, 2018, unpublished data) and provided guidance in planning the 1-year intervention, expected to start in autumn, 2018.

### Intervention

DSNs will be trained in using the SMASc questionnaire to score the SMS needs. They will also be trained in using and instructing participants how to use the app. Furthermore, they will be taught how to use the Web page, with diabetes facts and illness integration support, included in the project. All recommendations to patients will be based on patients’ stories and expressed needs as well as patients’ scores on the SMASc questionnaire to make the individual plans person-centered.

Both the intervention and control groups will continue with their usual diabetes care, including all medical visits, tests, and diabetes support programs. The starting point for the 4-months’ intervention is the baseline scoring on the SMASc questionnaire, resulting in a tailored person-centered plan for self-management through monitoring and interaction with DSN. The intervention group will receive the app, including instructions on how to use it, and the Web page with diabetes facts, as described above. DSNs will also assist in installing the app on the participants’ smartphone or tablet. The software can be personally tailored according to the participant’s needs to provide a personal overview of how food, exercise, medicine, blood sugar, and BP interact. The participants can evaluate food intake, blood glucose levels, insulin, medicine intake, exercise (physical activity), and weight over time and receive reminders if they would like them. Participants may, if they wish, choose whether their DSN should have access to their data or not. The system will maintain logs of all outgoing and incoming messages, and incoming blood glucose values will be graphed by the system, which individuals can view.

#### Diabetes Management App

The diabetes management app, mySugr, offers data tracking and coaching services for people living with type 1 or type 2 diabetes. The mySugr App is a registered certified medical device, and carries the CE marking (Medical Devices Directive 93/42/EEC). Furthermore, the app is registered with the Food and Drug Administration, and per se, it is required to meet the highest of data security and reliability standards. The app is available for iOS and Android operating systems for mobile access. There is easy manual input of data, which may be synchronized with selected glucose meters via Bluetooth, and one can easily log in and maintain a record of diabetes clinical data. The app uses these data to provide analysis and trending results. Currently, the app offers features for self-monitoring of diet, exercise, blood sugar, smoking, weight, and medication as well as gamification to support improved self-management. Reminders and self-reflection are linked to these areas, as are statistics and visualization [31-33].

#### Website

The website (www.t2d.se-in Swedish) is a complement to the app with opportunities for social support, factual information, and links for interaction with other patients through discussion forums as well as opportunities for interaction and support from the diabetes nurse via a messaging feature.

### Power Calculation and Sample Size

A power calculation showed that with a sample of 46 participants per group, a power of 80% with an alpha<.05 will demonstrate significant HbA_1c_ difference of 6 mmol/mol between the groups and a within-group SD assumed to be 9 [10]. This means that there is an 80% likelihood that the study will yield a statistically significant effect and allow us to conclude that the mean HbA_1c_ differs between the intervention group versus the control group. To compensate for dropouts, the study needs to enroll 46 participants per group for a total of at least 120 participants.

### Randomization and Blinding

Eligible participants will be randomized to either the intervention or the control groups in a 1:1 ratio. Owing to the nature of the intervention, participants will be aware of their treatment allocation. Therefore, the blinding of participants will not be possible. However, DSNs in charge at HCCs will not be involved in the randomization, preparation of the envelopes with study information, or statistical analyses.

### Study Population and Sampling

Sampling will be conducted both at the organizational HCC level and at the patient level. First, primary health care managers at HCCs in a county in northern Sweden have previously received verbal and written information on the purpose of the study and the implementation process as well as inclusion criteria, and all have accepted participation. After receiving consent from primary health care managers, DSNs at 6 HCCs accepted participation, got verbal and written information about the study, and were invited to participate in the co-design and participatory workshop and the RCT study and were further asked to collaborate in patient recruitment using a clustered RCT design.

#### Inclusion Criteria

**Figure 1:**
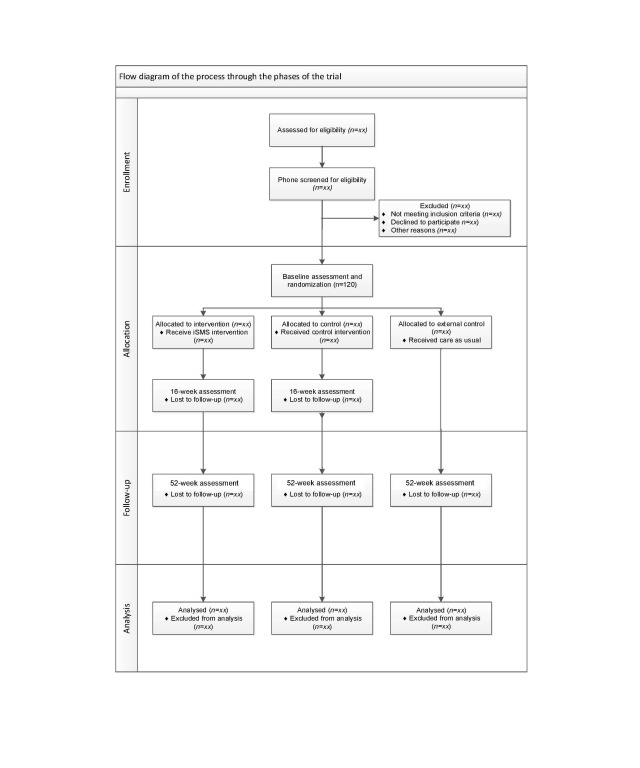
Flow diagram of trial design.

Eligible participants are adult, aged ≥18 years, patients with T2D diagnosed within the last 5 years, Swedish-speaking, noninsulin-treated at inclusion, and own a smartphone. They will be randomized to either an intervention group (n=46) or a control group (n=46). An external comparison group (n=46) will be recruited from 2 different HCCs to analyze for a possible Hawthorne effect. The recruitment process will start in September 2018. The study flow chart is presented in Figure 1.

#### Exclusion Criteria

The exclusion criteria are planned (within 2 years) or current pregnancy, life-threatening physical illness (eg, cancer), and cognitive impairment. Furthermore, patients not responsible for their care and those not residing in their home environment (eg, those in nursing homes and in-patient hospital wards) will be excluded. The recruitment process will build on patients identified in the electronic medical record system at each HCC cared for by DSNs. Participants will be contacted via phone by the research team to discuss the study and gain informed consent about the randomization process. Informed consent will be obtained from all participants before they are enrolled in the study.

#### Control Groups

The control groups will be included in the data collection of laboratory values as well as questionnaires, similar to the intervention group. In addition to this, they will receive the usual care and take part in a minimal intervention in the form of a brochure about self-management of diabetes.

#### Diabetes Specialist Nurses

The study includes DSNs (n=6-10) from the 6 HCCs. DSNs are fundamental to this RCT study because they will take part in introducing and training patients to use the app and recommend individualized self-care support from a website. The training of the nurses will involve learning to use the SMASc questionnaire, to develop the person-centered plan for SMS, and how to train the patients in using a medical software product.

### Outcome Assessments

At baseline and 4 and 12 months’ evaluation, assessments will be conducted. Baseline assessments will involve the collection of demographic data regarding age, gender, marital or family relationships, housing, education, and employment; self-reported outcome measures via questionnaires; and collection of laboratory tests and physical measurements via participants’ medical records. Finally, information regarding diabetes duration, tobacco use, prescribed medication, diet, and exercise habits will be collected. For the follow-up assessments, completion of self-reported outcome measures via questionnaires and collection of laboratory tests and physical measurements via participants’ medical records will be performed.

### Outcome Measures

#### Primary Outcomes

The primary outcome among patients is a change in glycemic control, measured as HbA_1c_ (in mmol/mol) by registered laboratories at baseline and 4 and 12 months’ follow-up. This data will be obtained from patient records. HbA_1c_ levels have been associated with an increased risk of diabetes-related complications. Therefore, the primary outcome in this RCT is the change in HbA_1c_. The main goals of glucose-lowering therapy in T2D are to reduce the risk of diabetes complications while minimizing harms associated with therapy, thus increasing both longevity and health-related QoL.

#### Secondary Outcomes

Secondary outcomes include metabolic measurements, BP, body mass index, waist circumference, and total and high-density lipoprotein cholesterol. Furthermore, lifestyle habits, physical activity, diet, smoking, changes in medical treatment, SMS, diabetes empowerment, diabetes-dependent QoL, improved illness perception, and improved eHealth literacy in the intervention group will be evaluated.

#### Instruments

##### Self-Management Assessment Scale

SMS needs will be measured by SMASc. This 10-item questionnaire has been developed within the research group and measures 5 domains, namely, knowledge, routines, goals, emotional support, and social support, rated on a 6-point Likert scale. Validity and reliability have been tested and found acceptable (manuscript, Öberg et al, 2018, unpublished data).

##### Audit of Diabetes-Dependent Quality of Life

Diabetes disease-specific QoL will be measured by the Audit of Diabetes-Dependent Quality of Life (ADDQoL) [34] at baseline and follow-up. This questionnaire measures patients’ perspectives on the impact of diabetes on their QoL in the following 19 domains: leisure activities, working life, journeys, holidays, physical health, family life, friendship and social life, personal relationship, sex life, physical appearance, self-confidence, motivation, people’s reactions, feelings about future, financial situation, living conditions, dependence on others, freedom to eat, and freedom to drink. It consists of 2 overview items, 1 assessing general overall QoL and the other the specific impact of diabetes on QoL [35,36]. Audit of Diabetes-Dependent Quality of Life has been shown to have good validity and reliability in research and practice [35,37]. In this RCT, the Swedish version, SE-ADDQoL, will be used.

##### Brief Illness Perception Questionnaire

Illness perception, measuring cognitive and emotional representations of diabetes, will be assessed using the Brief Illness Perception Questionnaire (IPQ) [38] at baseline and at follow-up. The instrument consists of a 9-item self-reported measure designed to assess cognitive and emotional representations of illness. The Brief IPQ measures concerns, consequences, emotions, identity, illness comprehensibility, personal and treatment control, and the timeline and causes of diabetes. In the Brief IPQ questionnaire, each item is rated using an 11-point Likert scale wherein higher scores indicate greater agreement with the item. The Brief IPQ has been shown to have good reliability and validity in research and practice [38]. In this RCT, the Swedish version, SE-B-IPQ, will be used.

##### European Health Literacy Survey Questionnaire

eHealth literacy will be measured by the shorter Swedish version of the European Health Literacy Survey Questionnaire. The Swedish version of European Health Literacy Survey Questionnaire will be used at baseline and follow-up [39,40]. The instrument consists of 16 items focusing on the following 4 dimensions of health literacy: the ability to access and obtain health information, ability to understand health information (not only in written form), ability to process and appraise health information, and ability to apply and use health information.

##### Electronic Health Literacy Scale

eHealth Literacy will also be measured by the eHealth Literacy Scale (eHEALS) [41]. The 8-item eHEALS scale will be tested and validated to assess consumers’ combined knowledge, comfort, and perceived skills at finding, evaluating, and applying electronic health information to health problems. In this study, a Swedish translated version of the eHEALS will be developed, and its psychometric properties will be tested.

##### Diabetes Empowerment Scale

Diabetes empowerment will be measured by the Diabetes Empowerment Scale (DES) [42]. The short-form Diabetes Empowerment Scale-Short Form, Swedish version will be used at baseline and follow-up. It includes 4 empowerment subscales: goal achievement, self-awareness, stress management, and readiness to change. A 5-point Likert scale is used. Originally, this questionnaire was based on SWE-DES-23, which is considered a valid and reliable tool to assess empowerment in diabetes and rheumatic disease [43,44]. SWE-DES-23 was tested and shortened to become Diabetes Empowerment Scale-Short Form, Swedish version, which was found to be valid and reliable in relation to the original version [43].

##### Intuitive Eating Scale

Eating behaviors will be measured by the Intuitive Eating Scale (IES) [45]. This 21-item scale measures the tendency to follow physical hunger and satiety cues when determining when, what, and how much to eat. In this RCT, the Swedish version, SWE-IES, will be used.

##### Health-related Quality of Life and Cost and Health Economic Evaluation

Health-related QoL and cost and health economic evaluation of the intervention will be measured by the EuroQol 5-Dimensional 5-Level Questionnaire (EQ-5D-5L) at baseline and follow-up. EQ-5D is a generic health-related QoL instrument and a standardized instrument for use as a measure of health outcome [46] from which a single-index value of the respondent’s health status can be derived. EQ-5D is commonly used to estimate the QoL components. Furthermore, it is possible to calculate quality-adjusted life years and thereby perform an economic evaluation of the intervention by means of quality-adjusted life years. It is also used as a health care performance indicator and in the measurement of population health in surveys [47,48]. EQ-5D-5L is a further development of EQ-5D and is based on a health profile consisting of a descriptive system and the EQ visual analog scale. The descriptive system consists of the following 5 dimensions: mobility, self-care, usual activities, pain or discomfort, and anxiety or depression. Each dimension has the following 5 severity levels: no problems, slight problems, moderate problems, severe problems, and extreme problems [49]. In this RCT, the Swedish version, SE-EQ-5D-5L, will be used.

### Data Analyses

All analyses to evaluate change over time with regard to intervention outcomes will be made with the intention-to-treat principle, which means that all participants are analyzed according to the group they were randomized into [50]. Appropriate imputation methods will be applied to the missing data.

Baseline and sociodemographic characteristics will be summarized using descriptive statistics. Continuous variables will be summarized as numbers of observed values and mean (SD) or median and quartiles when appropriate. Categorical variables will be described using central tendencies and variability. Differences between groups will be analyzed using inferential statistics.

### Ethical Considerations

This study will conform to the principles of the declaration of Helsinki [51]. Ethical approval for this trial was granted by the Regional Ethical Review Board at Umeå University (Dnr 2014-179-31M). The major ethical considerations for this study concern the data collection, which might be experienced as tiresome for the participants. However, this risk for the participants is judged as relatively small in comparison with the benefits of receiving person-centered SMS.

## Results

This trial is currently open for recruitment. The anticipated completion date for the study is September 2019.

## Discussion

### Intervention Design

This study protocol describes a planned project aiming to develop and implement an intervention consisting of person-centered, iSMS in primary health care for people with T2D and to evaluate its effectiveness. An intervention like this, in which patients and health care providers are involved in the developmental phase, can lead to more effective SMS and sustainable longer-term effects on health among patients.

The design for this intervention is based on experiences in the research group from previously conducted focus group interviews with primary health care nurses, individual interviews with patients with T2D, a multistakeholder workshop, and results from other studies [19,20,52,53]. Merging current research is beneficial to develop clinically useful interventions based on theory, which could be tailored more specifically for the participants through a cocreative design [54].

Any modifications to the study protocol will be discussed and agreed upon by consensus between the research group before implementing them, and all changes will be documented in a memorandum.

### Conclusions

This study, with its focus on iSMS, will provide insights regarding suitable ways to promote and develop a person-centered intervention regarding the usage of mobile tools. If successful, the intervention has the potential to become a model for the provision of SMS to people with T2D.

## Abbreviations

ADDQoL: Audit of Diabetes-Dependent Quality of Life
BP: blood pressure
DES: Diabetes Empowerment Scale
DSN: diabetes specialist nurse
eHealth: electronic health
eHEALS: eHealth Literacy Scale
EQ-5D-5L: EuroQol 5-Dimensional 5-Level Questionnaire
HbA_1c_: glycated hemoglobin
HCC: health care center
IES: Intuitive Eating Scale
IPQ: Illness Perception Questionnaire
iSMS: interactive self-management support
PCC: person-centered care
QoL: quality of life
RCT: randomized controlled trial
SMASc: Self-Management Assessment Scale
SMS: self-management support
T2D: type 2 diabetes
